# Critical Role of Maternal Selenium Nutrition in Neurodevelopment: Effects on Offspring Behavior and Neuroinflammatory Profile

**DOI:** 10.3390/nu14091850

**Published:** 2022-04-28

**Authors:** Maria Antonietta Ajmone-Cat, Roberta De Simone, Anna Maria Tartaglione, Antonella Di Biase, Rita Di Benedetto, Massimo D’Archivio, Rosaria Varì, Laura Ricceri, Federica Aureli, Francesca Iacoponi, Andrea Raggi, Francesco Cubadda, Susan J. Fairweather-Tait, Gemma Calamandrei, Luisa Minghetti

**Affiliations:** 1 National Center for Drug Research and Evaluation, Istituto Superiore di Sanità, 00161 Rome, Italy; roberta.desimone@iss.it; 2 Center for Behavioral Sciences and Mental Health, Istituto Superiore di Sanità, 00161 Rome, Italy; annamaria.tartaglione@iss.it (A.M.T.); laura.ricceri@iss.it (L.R.); gemma.calamandrei@iss.it (G.C.); 3 Department of Food Safety, Nutrition and Veterinary Public Health, Istituto Superiore di Sanità, 00161 Rome, Italy; antonella.dibiase@iss.it (A.D.B.); rita.dibenedetto@iss.it (R.D.B.); federica.aureli@iss.it (F.A.); francesca.iacoponi@iss.it (F.I.); andrea.raggi@iss.it (A.R.); francesco.cubadda@iss.it (F.C.); 4 Center for Gender-Specific Medicine, Istituto Superiore di Sanità, 00161 Rome, Italy; massimo.darchivio@iss.it (M.D.); rosaria.vari@iss.it (R.V.); 5 National Centre for the Control and Evaluation of Medicines, Istituto Superiore di Sanità, 00161 Rome, Italy; 6 Norwich Medical School, University of East Anglia, Norwich NR4 7UQ, UK; s.fairweather-tait@uea.ac.uk; 7 Research Coordination and Support Service, Istituto Superiore di Sanità, 00161 Rome, Italy; luisa.minghetti@iss.it

**Keywords:** selenium, perinatal exposure, diet, neuroinflammation, microglia, behavior, oxidative stress

## Abstract

Research in both animals and humans shows that some nutrients are important in pregnancy and during the first years of life to support brain and cognitive development. Our aim was to evaluate the role of selenium (Se) in supporting brain and behavioral plasticity and maturation. Pregnant and lactating female rats and their offspring up to postnatal day 40 were fed isocaloric diets differing in Se content—i.e., optimal, sub-optimal, and deficient—and neurodevelopmental, neuroinflammatory, and anti-oxidant markers were analyzed. We observed early adverse behavioral changes in juvenile rats only in sub-optimal offspring. In addition, sub-optimal, more than deficient supply, reduced basal glial reactivity in sex dimorphic and brain-area specific fashion. In female offspring, deficient and sub-optimal diets reduced the antioxidant Glutathione peroxidase (GPx) activity in the cortex and in the liver, the latter being the key organ regulating Se metabolism and homeostasis. The finding that the Se sub-optimal was more detrimental than Se deficient diet may suggest that maternal Se deficient diet, leading to a lower Se supply at earlier stages of fetal development, stimulated homeostatic mechanisms in the offspring that were not initiated by sub-optimal Se. Our observations demonstrate that even moderate Se deficiency during early life negatively may affect, in a sex-specific manner, optimal brain development.

## 1. Introduction

Over the past 30 years, substantial attention has been given to the influence of nutrition during critical developmental windows on offspring health and chronic disease risk later in life, a concept known as the Developmental Origins of Health and Disease hypothesis (DOHaD) [1,2]. Epidemiological and animal model studies have demonstrated that an adequate diet during pregnancy and lactation allows appropriate supply of the micronutrients necessary to support rapidly developing brain systems and maturation of cognitive functions [3,4,5]. Thus, maternal nutritional imbalance can permanently affect offspring’s physiological and neurodevelopmental outcomes [6].

An important micronutrient that regulates development, growth, and differentiation is the trace element selenium (Se). Unlike other essential trace elements with biological roles in proteins in the form of cofactors, Se is co-translationally incorporated into the polypeptide chain as part of the amino acid selenocysteine to form selenoproteins. There are 25 identified selenoproteins [7] and they are involved in diverse homeostatic processes, including brain function, anti-oxidant regulation, thyroid hormone metabolism, and immune function [8,9].

Brain function-associated selenoproteins include: the glutathione peroxidases (GPxs) and thioredoxin reductases (TXNRDs), which are responsible for much of the antioxidant effect of Se; selenoprotein P (SelP), which has a special role in delivering Se to neurons [10,11]; and selenoprotein W (SelW) with mainly anti-oxidant properties [8]. These proteins are involved in diverse functions, including motor performance, coordination, memory, and cognition [10,12]. Evidence suggests that Se may play a role during critical periods of brain development. For example, neuron-specific glutathione peroxidase 4 (GPx4) depletion caused ataxic gait and hyperexcitable phenotype in mice as early as the first few weeks of life [13]. SelP knockout mice exhibited severe neurological dysfunctions at weaning that were largely prevented when mice were fed an Se-enriched diet [14]. Mice with targeted deletion of thioredoxin reductase 1 (Txnrd1) in the nervous system displayed growth retardation and movement disorders, suggesting a specific role of Txnrd1 in brain development and function [15]. Transgenic studies showed that disruption of selenoprotein synthesis leads to the loss of the cortical and hippocampal gamma-aminobutyric acid (GABA)ergic parvalbumin-immunoreactive (PA+) interneurons [16].

As far as dietary reference values are concerned, the European Food Safety Authority (EFSA) set an adequate intake (AI) of 70 μg Se/day for adults [17]. Taking into account adaptive changes in Se metabolism that occur during gestation, the AI for adult women applies to pregnancy, whereas for lactating women the AI for Se was increased to 85 μg/day, which includes an additional 15 μg/day to provide Se secreted in breast milk [17]. The environmental distribution of Se varies considerably worldwide, with the Se content of foods depending on the Se content of the soil and the soil’s geochemistry. The different Se compounds present in foods add further complexity with respect to intake data, with l-selenomethionine being the major chemical species found in the diet [18]. Dietary intakes of Se are high in countries such as Venezuela, Canada, the USA, and Japan, and much lower in Europe (in particular in Eastern Europe) [17,19].

Maternal Se status is crucial for proper fetal development and Se deficiency during pregnancy increases the risk of pregnancy complications, including fetal growth restriction [20]. In addition, evidence exists that prenatal Se status may affect children’s psychomotor, language, and cognitive development [21,22]. Studies from animal models have demonstrated that Se maternal status strongly influences the metabolic profile, the innate and adaptive immune responses, as well as the reproductive system of the progeny [20,23,24] (and references therein). However, the effects of maternal Se nutrition on behavioral responses, neuroinflammation, and oxidative stress have not yet been comprehensively characterized. In addition, there have been only a few studies aimed at analyzing sex-dependent vulnerability to different inadequate maternal Se intake. Thus, in the present work, pregnant and lactating female rats and their offspring until juvenile age were fed isocaloric diets differing in Se content (Se-optimal, Se-suboptimal, and Se-deficient diet) providing either adequate, suboptimal or inadequate intake [25]. Neurodevelopmental and neuroinflammatory markers, brain, and liver anti-oxidant enzymatic activity were then analyzed in offspring of both sexes.

## 2. Materials and Methods

### 2.1. Animals

**Figure 1:**
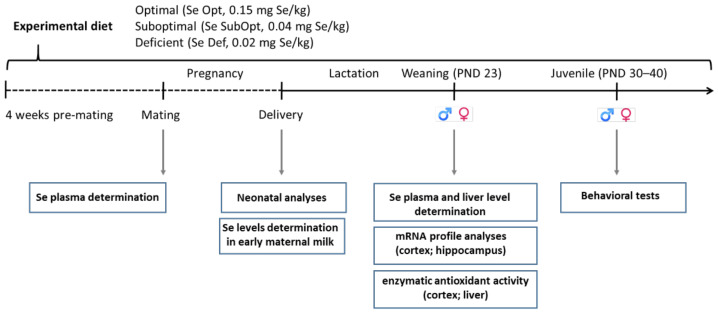
Experimental timeline. Female rats were fed an optimal (0.15 mg/kg), suboptimal (0.04 mg/kg) or deficient (0.02 mg/kg) Se diet from four weeks pre-mating until the end of lactation. At weaning, offspring were fed the same diet as their respective dams until completion of the behavioral assessment at PND 40. Neonatal analyses, plasma and tissue collection for Se level determination, biochemical, molecular analyses, and juvenile behavioral tests were performed at the time points indicated. Se, selenium; Se Opt, Se-optimal; Se SubOpt, Se-suboptimal; Se De, Se-deficient; PND, postnatal day; ♀, females; ♂, males.

Wistar rats were kept under standard animal housing (temperature 20 ± 2 °C; humidity 60–70%) with food and water ad libitum, under a 12 h–12 h light/dark cycle (lights on from 7:00 a.m. till 7:00 p.m.). Following the adaptation period, adult female rats were assigned to one of the three experimental groups based on different Se dietary content (as l-selenomethionine), namely 0.15 mg/kg (Se-optimal, Se Opt diet), 0.04 mg/kg (Se-suboptimal, Se SubOpt diet) and 0.02 mg/kg (Se-deficient, Se Def diet), four weeks prior to mating and through pregnancy and lactation. At weaning, i.e., at postnatal day (PND) 23, offspring were fed the same diet as their respective dams until completion of the behavioral assessment at PND 40 (Figure 1).

Female rats were mated with males (2:1) for 4 to 5 days to cover the duration of an estrous cycle. Day of birth was designated as PND 0. On PND 1, litters were culled to equal numbers to standardize litter size, with the aim to have ten pups, sex balanced, per litter. At weaning (PND 23), male and female offspring were separated and housed two/three per cage up to the end of all experiments.

### 2.2. Diets

Rat diets were prepared by Envigo (Udine, Italy) by supplementing a selenium-deficient diet (Teklad Custom Diet TD.92163), containing <0.01 mg Se/kg with l-selenomethionine to obtain a diet with optimal (Se Opt, 0.15 mg Se/kg), suboptimal (Se SubOpt, 0.04 Se mg/kg) or inadequate (Se Def, 0.02 Se mg/kg) Se content, respectively.

### 2.3. Plasma and Milk Sampling

Blood samples were collected in ethylenediaminetetraacetic acid (EDTA) (0.2 mg/100 mL) from the left atrium of the heart of rats anesthetized by isoflurane. Plasma samples—obtained after centrifugation—were aliquoted and stored at −80 °C until Se determination. Early milk was recovered from the stomachs of pups (PND 1) and stored at −80 °C until Se levels determination.

### 2.4. Brain and Liver Sampling

On PND 23, in order to collect brain samples, male and female rats in each experimental group were rapidly decapitated and brains were immediately sectioned on ice to obtain cortex and hippocampus (*n* = 10 rats per experimental group). Brain samples were flash-frozen and stored at −80 °C until mRNA expression analyses. Livers were immediately excised, weighed, and divided into smaller pieces and stored at −80 °C for enzymatic analysis or Se determination.

### 2.5. Selenium Determination

For determination of Se levels we analyzed: (i) plasma from 5 pre-mating females for each group; (ii) early milk from stomachs of 6 (from Se Opt group), 5 (from Se SubOpt group) and 11 (from Se Def group) pups at PND 1; (iii) liver from 8 (5 males and 3 females) for Se Opt group, 7 (4 males and 3 females) for Se SubOpt group, 8 (5 males and 3 females) rats for Se Def group at weaning (PND 23); (iv) plasma from 5 rats for each group at weaning (PND 23).

The quantitative analysis of total Se was carried out with external calibration by means of an 8800 Triple Quad ICP mass spectrometer from Agilent Technologies (Tokyo, Japan). Plasma was diluted 1:50 with 0.5% *v*/*v* nitric acid (HNO_3_). Milk and livers were digested in a microwave system with HNO_3_ and hydrogen peroxide (H_2_O_2_). Oxygen was used as a reaction gas for mass shift (selenium dioxide ion (SeO^+^)) in inductively coupled plasma-mass spectrometry/mass spectrometry (ICP-MS/MS) determinations and m/z 94 and 96 were resorted to as analytical masses. Accuracy was checked by the reference materials Seronorm Serum L1, NIST 1549 (Non-Fat Milk Powder), BCR 063R (Skin Milk), and NIST 1577c (Bovine liver). A detailed description of the analytical procedures is given in the Supplementary Materials S1 and Table S1 therein.

### 2.6. Behavioral Testing

At the juvenile stage, female and male rats were assessed in the following behavioral tests: Open-Field (OPF, PND 30), Spontaneous alternation (Y-maze, PND 35), Light/Dark test (PND 38), Rotarod test (PND 40).

The number of animals undergoing behavioral testing for each group was the following: 12 rats (6 males and 6 females) for Se Opt group, 14 (7 males and 7 females) for Se SubOpt group, and 13 (7 males and 6 females) for Se Def group.

All apparatuses were cleaned with 70% alcohol following each animal testing. All behavioral procedures were carried out between 9:00 a.m. and 2:00 p.m.

#### 2.6.1. Open-Field (OF)

To assess locomotor activity and exploration of a novel environment, rats were tested in the OF test at PND 30. The OF apparatus consisted of a black Plexiglas box (80 × 80 × 60 cm). Each subject was placed in one corner of the apparatus and the spontaneous locomotor activity of the animals was video-recorded for 10 min (as described in [26]). Distance traveled and mean velocity were analyzed using ANY-Maze video-tracking software (Stoelting Europe, Dublin, Ireland).

#### 2.6.2. Y-Maze

To assess the spatial working memory and exploratory activity, rats were tested in the Y-maze test at PND 35. The apparatus consisted of three identical arms (50 × 16 × 32 cm) diverging at an angle of 120° one to the other and an equilateral triangular central area. Each animal was placed in the center of the Y-maze and was free to explore the arena for 8 min. The following dependent variables were registered and analyzed by The Observer XT behavioral coding software (Noldus, Wageningen, The Netherlands): the total number of arm entries and the sequential list of arms entered to assess the number of alternations. An arm entry was scored when the rat placed the four paws within that arm. An alternation was defined as an entry into three different arms on consecutive choices. Spontaneous alternation was calculated using the following formula: (number of alternations/total number of entries − 2) × 100.

#### 2.6.3. Light/Dark Test

To assess the anxiety-like behavior, rats were tested in the Light/Dark test at PND 38. The apparatus consisted of an opaque Plexiglas box with smooth walls and floor (70 × 30 × 35 cm), subdivided into two chambers by a partition possessing a doorway through which rats could traverse. One compartment had white walls and floor, whereas the other one had black walls and floor. The black compartment was not illuminated and covered by a black ceiling, while the white compartment had no ceiling and was intensely illuminated by a bright white light (100 W). Each animal was placed in the light chamber and was free to explore the arena for 5 min. Latency to enter the light from the dark compartment and time spent in each compartment were analyzed by The Observer XT behavioral coding software (Noldus, Wageningen, The Netherlands).

#### 2.6.4. Rotarod Test

To assess the motor coordination and balance, rats were tested in the Rota-Rod apparatus (model 47700, Ugo Basile, Germonio, Italy) at PND 40. All rats underwent a two-day training to walk against the motion of a rotating drum at a constant speed of 12 R.P.M (rotations per minute) for a maximum of 2 min. Following the training days, a one-day test of three trials was performed using an accelerating speed level (4 to 40 R.P.M) mode of the apparatus over 5-min. The mean latency to fall off the rotarod was recorded.

### 2.7. Real-Time Quantitative Polymerase Chain Reaction (RT-PCR)

Dissected cortexes and hippocampi from 10 rats (5 males and 5 females) for each group were homogenized in Trizol Reagent (Sigma, St. Louis, MO, USA) and mRNA extraction was performed on supernatants. Total RNA (1 µg) from each sample was transcribed into complementary DNA using the High-Capacity cDNA Reverse Transcription Kit (Applied Biosystems, Thermo Fisher Scientific, Waltham, MA, USA), according to the manufacturer’s instructions. Real-time Polymerase Chain Reaction (PCR) was performed on the reverse transcription products with TaqMan master mix to analyze the relative transcript levels of the macrophage/microglial marker cluster of differentiation (CD)11b, the astrocyte marker glial fibrillary acidic protein (GFAP), arginase-1 (Arg-1), and β-actin, or SYBR™ Green master mix (Applied Biosystems, Thermo Fisher Scientific, Waltham, MA, USA) for interleukin-1β (IL-1β) and inducible nitric oxide synthase (iNOS), using an ABI Prism 7500 Sequence Detection System (Applied Biosystems, Foster City, CA, USA).

TaqMan primers for CD11b (Acc. Number NM_012711.1), GFAP (NM_017009.2) and Arg-1 (Acc. Number NM_007482.3), and β-actin (Acc. Number NM_031144.3) were TaqMan™Gene Expression Assay (Applied Biosystems, ThermoFisher Scientific, Waltham, MA, USA). PrimeTime^®^ Predesigned Quantitative Polymerase Chain Reaction (qPCR) Assays for IL-1β (Acc. Number: NM_031512(1) and iNOS (NM_012611) were from Integrated DNA Technologies (IDT, TEMA Ricerca, Bologna, Italy). All samples were run in duplicate, and each PCR well contained 20 μL as a final volume of reaction, including 2 μL complementary DNA corresponding to approximately 60 ng total RNA, 750 nM of each primer, and 10 μL PCR master mix. Thermal cycling conditions were as follows: 1 cycle at 95 °C for 10 min, 40 cycles at 95 °C for 15 s, and 60 °C for 1 min. The relative expression level of each mRNA was calculated using the 2^−ΔΔCt^ method, normalized to β-actin, and relative to the control group (i.e., male rats of the Opt Se dietary group).

### 2.8. Glutathione Peroxidase Enzymatic Activity

Enzymatic antioxidant activity of GPx was evaluated in brain cortex and liver samples from 10 rats (5 males and 5 females) for each group by commercial kits (Cayman, Ann Arbor, MI, USA), following the manufacturer’s instructions. Cumene hydroperoxide was used as a substrate, so we mainly measured GPx-1 activity.

### 2.9. Statistical Analyses

Behavioral, molecular and GPx activity data were analyzed by two way-analysis of variance (2 way-ANOVA) with diet and sex as between-factors followed by post hoc Tukey’s analysis on significant effects using the GraphPad Prism version 8.3.1 (GraphPad Software, San Diego, CA, USA). Se levels were analyzed by the Kruskal-Wallis’ test followed, when significant, by a Dunn’s post hoc test for multiple comparisons, using the SPSS v.27 software (IBM, Armonk, NY, USA). A *p* value < 0.05 (two-tailed) was considered statistically significant.

## 3. Results

### 3.1. Selenium Levels in Dams and Offspring

Plasma Se, a reliable biomarker of Se intake when selenomethionine is the dietary Se form, was investigated in dams pre-mating; the levels were not significantly different among the three groups (Se Opt, SubOpt, and Def) (Table 1). Se content in early maternal milk, the most useful biomarker of offspring nutrition at delivery, revealed that the offspring of the Se SubOpt and Se Def groups experienced poor Se nutrition at birth, being the Se content about 1/3 of that measured in the Se Opt group (Table 1).

Liver Se levels in offspring at weaning (PND 23) (Table 2) revealed that Se stores were severely depleted in both Se Def and SubOpt groups as compared to Opt group, in which liver Se levels were >5 times higher (Table 2). Liver Se was mirrored by plasma Se in offspring at PND 23 (Table 3).

### 3.2. Effect of Diet on Reproductive Performance and Pup Somatic Growth

We did not observe overt detrimental effects of Se SubOpt or Def diets on reproductive performance as evaluated by number of delivered pups (Mean ± standard deviation (SD), Opt = 12.8 ± 4.6; SubOpt = 11.8 ± 3.5, Def = 12.0 ± 4.3), sex ratio (Mean ± SD, Opt = 1.0 ± 0.2; SubOpt = 0.7 ± 0.2, Def = 0.9 ± 0.7), and body weight of pups at birth (Mean ± SD, Opt = 6.8 ± 0.8; SubOpt = 6.8 ± 1.2, Def = 7.3 ± 1.9).

Similarly, there was no significant effect of Se SubOpt or Def diets when considering offspring somatic growth, namely body weight and body length, at any of the time points assessed from PND 4 to PND 12 (Supplementary Materials Figure S1).

### 3.3. Effects of Maternal Se Intake on Behavioral Responses in Juvenile Offspring

**Figure 2:**
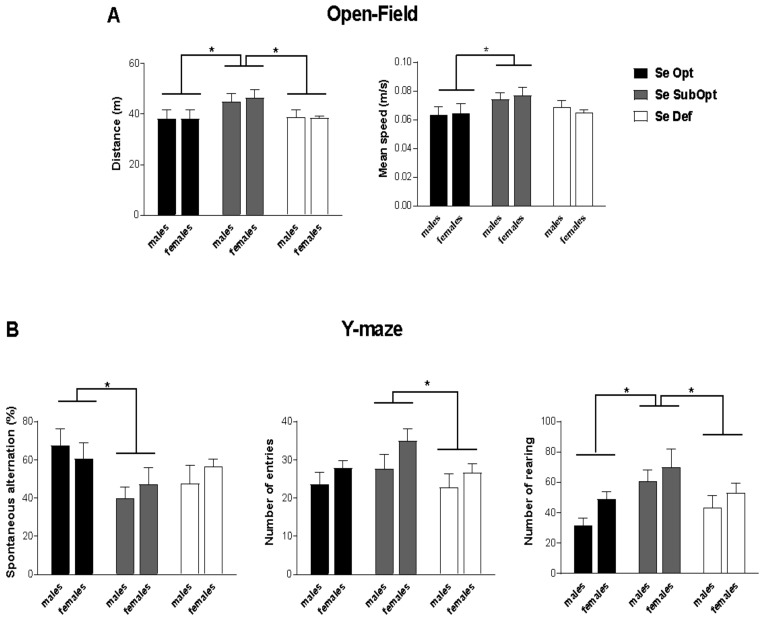
Open-Field and Y-maze test in juvenile rats. Juvenile rats of both sexes experiencing a dietary regimen with suboptimal Se content showed a hyperactive profile when exploring a novel environment (**A**) and exhibited perseverative behavior (alternated less) and elevated explorative activity in Y-maze (**B**). Se Opt: *n* = 6 males (M), 6 females (F); Se SubOpt: *n* = 7M, 7F; Se Def: *n* = 7M, 6F. Data are represented as mean ± standard error of the mean (SEM). * *p* < 0.05.

At PND 30, Se SubOpt diet impacted locomotor activity during exploration of the novel environment (open-field test) measured by total distance (*p* < 0.05) and mean speed (*p* < 0.05). Specifically, Se SubOpt rats of both sexes traveled longer distances and moved with higher mean velocity compared to either Se Def or Se Opt rats (*p* < 0.05 after post hoc comparisons, Figure 2A).

In the exploration of Y-maze, Se SubOpt rats showed higher frequency of rearing (*p* < 0.05 vs. Se Def, *p* < 0.01 vs. Se Opt), performed higher number of entries than Se Def rats (*p* < 0.05) together with a lower percentage of spontaneous alternation among the three arms of the maze (*p* < 0.05 vs. Se Opt rats, Figure 2B). These findings suggest a perseverative and hyperactive behavioral profile in Se SubOpt rats.

At PND 38–40, analysis of anxiety responses (Light/Dark test) and motor coordination (Rota-Rod test) did not reveal any detrimental effects of Se Def or Se SubOpt diets or their interaction with sex (Supplementary Materials Figure S2).

### 3.4. Effects of Maternal Se Intake on Brain Immune Profile in Juvenile Rats

To characterize the effects of different maternal Se dietary intakes on the brain neuroinflammatory profile of the offspring, we analyzed the mRNA expression levels of the microglia/macrophage activation marker CD11b, the astrocytic activation marker GFAP, the inflammatory cytokine IL-1β, and the inflammatory-oxidative stress-related enzymes iNOS and Arg-1, in the cortex and hippocampus of male and female rats sacrificed at weaning (PND 23).

We focused our analyses on these genes since, in addition to their central role in the inflammatory response, they also play key roles in brain development, plasticity, and homeostasis.

The relative levels of their mRNA in each experimental group were expressed as the fold change versus the levels found in the corresponding male’s Se Opt group, taken as 1, to assess sex-related differences, Se-dependent effects, and their possible interaction.

**Figure 3:**
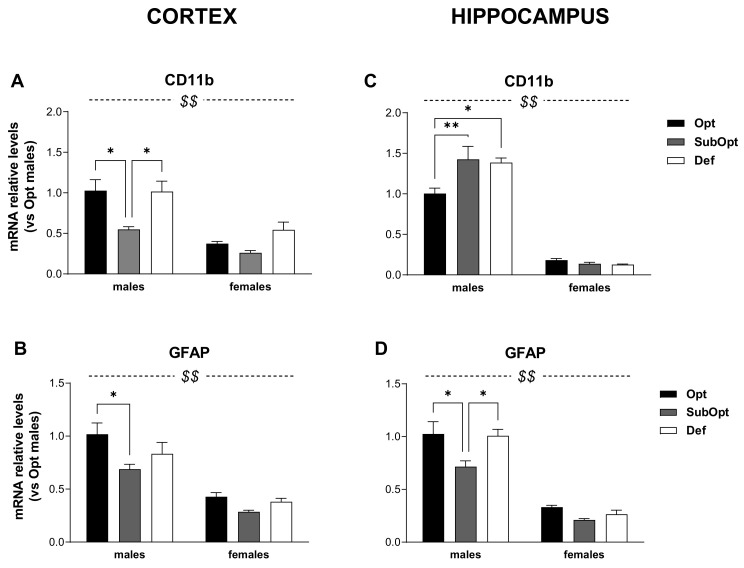
Effects of different Se supply on the level of CD11b and GFAP mRNA in the offspring’s cortex and hippocampus, evaluated by real-time PCR. The relative expression level of each mRNA was calculated using the 2^−ΔΔCt^ method, normalized to β-actin, and relative to the control group (i.e., male rats of the Opt Se dietary group). Each bar represents the mean ± SEM (*n* = 5M, 5F). Cortex (**A**,**B**) ^$$^ *p* < 0.0001 females vs. males (CD11b: *p* = 0.001 for Se Opt females vs. males; *p* = 0.015 for Se Def females vs. males. GFAP: *p* < 0.0001 for Se Opt females vs. males; *p* = 0.005 for Se SubOpt females vs. males; *p* = 0.001 for Se Def females vs. males; Tukey’s post hoc test); * *p* < 0.05. Hippocampus (**C**,**D**) ^$$^ *p* < 0.0001 females vs. males (*p* < 0.001 for female vs. male rats within all dietary groups, for both genes; Tukey’s post hoc test); * *p* < 0.05, ** *p* < 0.01. CD11b, cluster of differentiation 11b; GFAP, glial fibrillary acidic protein; PCR, Polymerase Chain Reaction.

**Figure 4:**
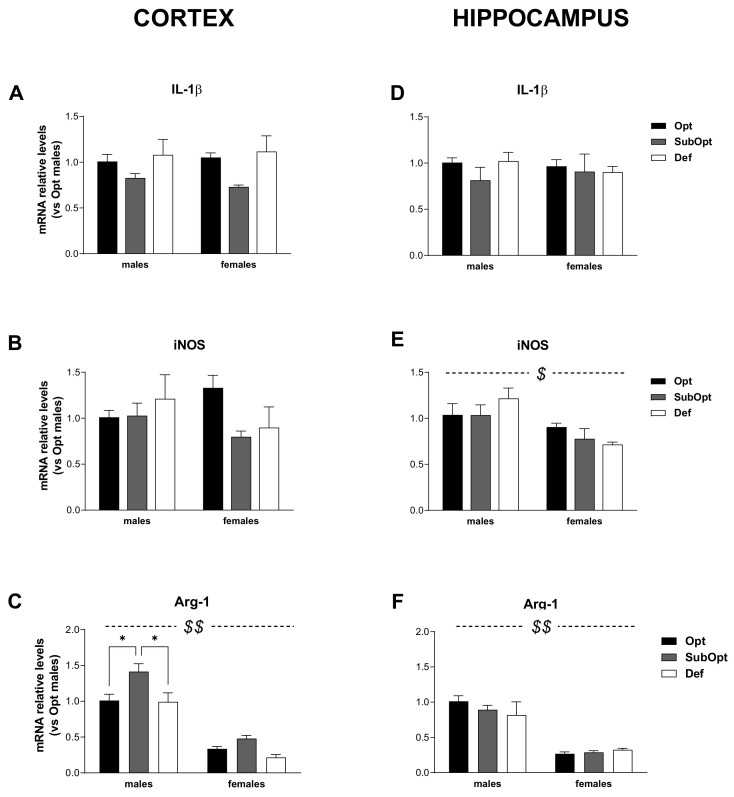
Effects of different Se supply on the level of IL-1β, iNOS, and Arg-1 mRNA in the offspring’s cortex and hippocampus, evaluated by real-time PCR. The relative expression level of each mRNA was calculated using the 2^−ΔΔCt^ method, normalized to β-actin, and relative to the control group (i.e., male rats of the Opt Se dietary group). Each bar represents the mean ± SEM (*n* = 5M, 5F). Cortex (**A**–**C**) ^$$^ *p* < 0.0001 females vs. males (Arg-1: *p* = 0.001 for Se Opt females vs. Se Opt males; *p* < 0.0001 for either Se SubOpt or Se Def females vs. Se SubOpt or Se Def males, Tukey’s post hoc test). * *p* < 0.05. Hippocampus (**D**–**F**) ^$^ *p* < 0.005 and ^$$^ *p* < 0.0001 for females vs. males (iNOS: *p* < 0.05 for Se Def females vs. Def males; Arg-1: *p* < 0.0001 for Se Opt females vs. Opt males; *p* < 0.005 for either Se SubOpt or Se Def females vs. Se SubOpt or Se Def males, Tukey’s post hoc test). IL-1β, interleukin-1β; iNOS, inducible nitric oxide synthase; Arg-1, arginase-1.

As shown in Figure 3 and Figure 4, the glial functional profile was altered by Se dietary intake in region-specific and sex-dimorphic manners.

In the cortex (Figure 3A,B), the levels of CD11b and GFAP were lower in females compared to males (sex main effect: *p* < 0.001). Moreover, Se SubOpt rats showed the lowest mRNA levels of both genes (diet main effect: *p* < 0.05 for CD11b; *p* < 0.01 for GFAP). Specifically, Se SubOpt males showed lower CD11b levels compared to Se Opt and Se Def males, and lower GFAP mRNA level compared to Se Opt males; the reduction did not reach significance in females (significant differences by Tukey’s post hoc analysis are reported in the figure legend). Se Def diet did not affect either of the two genes in this brain area.

Also in the hippocampus (Figure 3C,D), we found a lower expression of CD11b and GFAP transcripts in females as compared to males (sex main effect: *p*< 0.0001).

However, the regulation of CD11b was opposite to that observed in the cortex and 2-way ANOVA revealed a sex × diet interaction (*p* < 0.01, Figure 3C), with Se SubOpt and Se Def diets increasing CD11b mRNA levels only in males.

The effects of Se diets on hippocampal GFAP expression (Figure 3D) were instead similar to that observed in cortex (diet main effect: *p* < 0.01), with Se SubOpt rats as a whole showing the lowest levels. Post hoc analysis revealed significance only for Se SubOpt males compared to Se Opt and Se Def males, while the effect did not reach significance in females.

When analyzing IL-1β mRNA levels (Figure 4A–D), they were comparable in males and females in both brain regions. A main effect of diet was found only in the cortex (*p* < 0.05), with Se SubOpt groups as a whole bearing reduced levels compared to the other groups (Figure 4A).

Regarding iNOS expression (Figure 4B–E), 2-way ANOVA did not yield significant sex- or diet- effects in the cortex (Figure 4B). In the hippocampus (Figure 4E), a main effect of sex was found (*p* < 0.01) with Se Def females showing lower iNOS levels than Se Def males.

As concerning Arg-1 mRNA (Figure 4C–F), 2-way ANOVA yielded a significant effect of sex in both brain regions (*p* < 0.0001), with females, bearing lower Arg-1 levels than males. In the cortex (Figure 4C) Se SubOpt groups showed higher Arg-1 levels than Se Opt and Se Def groups (diet main effect: *p* < 0.01). The increase reached significance only in males. In the hippocampus, Arg-1 mRNA levels were not significantly affected by the diet.

In brief, these data demonstrate a basal sex-specific profile of expression for most of the genes analyzed, with males showing higher levels of CD11b, GFAP, and Arg-1 in both cortex and hippocampus, and higher iNOS levels in the hippocampus. In addition, in line with behavioral observations, the data demonstrate that suboptimal rather than deficient Se intake alters the glial functional profile of rats at weaning, with prominent effects occurring in males, mostly in the cortex.

### 3.5. Effects of Maternal Se Intake on GPx Activity in Brain Cortex and Liver of Juvenile Rats

GPx enzymes are a major class of functionally important selenoproteins. GPx activity was measured in the cortex and liver of male and female offspring at PND 23.

**Figure 5:**
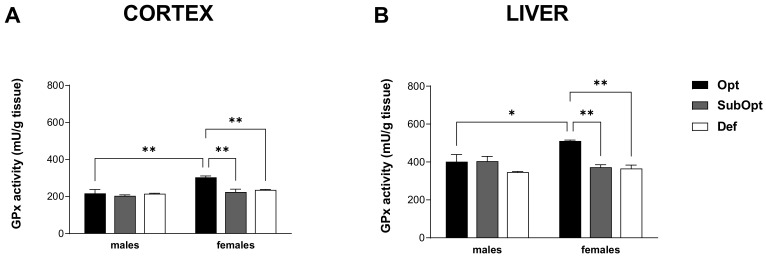
Effects of different Se supply on the enzymatic antioxidant activity of GPx in the offspring’s cortex (**A**) and liver (**B**). Each bar represents the mean ± SEM (*n* = 5M, 5F). * *p* < 0.05, ** *p* < 0.01 (Tukey’s post hoc test). GPx, glutathione peroxidase.

2-way ANOVA revealed a significant sex × diet interaction in both tissues (*p* < 0.05). Specifically, both Se SubOpt and Se Def diets reduced GPx activity in females, but not males, in the two tissues. The higher activity levels found in Se Opt females compared to Se Opt males, in both tissues (cortex: *p* < 0.01 and liver: *p* < 0.05), were thus abrogated by Se SubOpt and Se Def intake (Figure 5A,B).

These results suggest that cortical and hepatic GPx activity in females, which is higher than in males in optimal conditions as already described [27], is at the same time more susceptible to Se deficiency.

## 4. Discussion

To the best of our knowledge, this is the first study on the effects of maternal selenium dietary intake during pregnancy and lactation on offspring considering brain inflammatory and anti-oxidant biomarkers and neurobehavioral development. Moreover, this study included both female and male offspring to unveil potential sex-dependent vulnerability. We show here that Se intake during prenatal and early life phases is critical for the expression of key inflammatory mediators in the cortex and hippocampus as well as for liver and cortex GPx anti-oxidant activity, and influences exploratory activity at the juvenile life stage.

The molecular characterization of the expression profile of inflammatory and plasticity-related genes in the cortex and hippocampus unveiled a greater vulnerability of male offspring to poor maternal Se nutrition than females. Brain-region-specific effects were also evidenced by our data, being the cortex highly affected by a suboptimal intake of Se and unaffected by a deficient Se diet intake, while the hippocampus was only moderately affected by both conditions, at least at the developmental stage examined (PND 23).

Specifically, suboptimal Se intake was associated with downregulation of CD11b and GFAP expression, and upregulation of Arg-1 in male cortex, while in females these effects were not significant. In males’ hippocampus only the downregulation of GFAP could be observed, accompanied by the upregulation of CD11b, also demonstrating a different vulnerability of the two brain regions examined. The effects of deficient Se intake were limited to the upregulation of CD11b in males’ hippocampus. The sexual dimorphic and region-specific vulnerability to suboptimal Se can reflect the emerging heterogeneity of glial population across sexes and brain regions [28,29]. The sexual dimorphism of astrocyte and microglia reactivity is increasingly viewed as a key factor determining the sex bias observed in many neurodevelopmental and neurological disorders, with males particularly vulnerable to life-long illnesses of neurodevelopmental origin [28,30,31,32,33,34,35].

Sex-specific effects of low Se intake have also been described in peripheral tissues of rodents and humans [36,37,38,39], with males typically being more adversely impacted by deviations in Se intake [40,41,42].

Interestingly, a very recent study in mice reported that the administration of a low Se diet (<0.05 mg/kg), four weeks prior to mating and throughout gestation, induced sexually dimorphic transcriptional changes of selenoprotein expression in maternal, fetal, and offspring peripheral tissues [43].

Our data indicate that suboptimal and deficient Se maternal nutrition reduced GPx activity in both liver and cortex of female but not male offspring, suggesting that a similar sex-specific mechanism of selenoprotein regulation can take place in the brain as in peripheral organs. In addition, the higher ability of the hippocampus to retain Se in case of depletion compared to the cortex [44], could underlie the different impacts of Se depletion on the two brain areas.

Though the mechanisms by which Se influences brain and behavior development are still to be fully elucidated, it is well known that the inflammatory markers found altered in the present study play a critical role in neural and glial plasticity as well as in neurogenesis [45]. Furthermore, selenoproteins, as GPxs, exert an important protective role in the brain against lipid peroxidation and oxidative stress, and modulate redox-sensitive transcription factors in both neurons and astrocytes (see [46] for review). The expression of some selenoproteins involved in the activation of the transcription factor nuclear factor kappa B (NF-kB), a master regulator of inflammatory and immune responses, has been found to be negatively correlated with Se status in murine leukocytes, mouse colon [47,48], and in colon biopsies from human healthy subjects [49]. More specifically, under suboptimal Se, their lower expression correlated with reduced activation of NF-κB and the related- immune and inflammatory signaling. A suboptimal selenium supply was also shown to down-regulate the expression of genes coding for non-selenoproteins, such as the kinase Glycogen synthase kinase 3 beta (Gsk3b), involved in the deactivation of the redox- and electrophile-sensitive transcription factor nuclear factor erythroid 2–related factor 2 (Nrf2) and of its related anti-inflammatory and antioxidant pathways [50]. Whether the same molecular mechanisms are triggered by suboptimal Se status in the brain is not clear, and it will deserve further investigation.

Overall, the transcript regulations observed in our study suggest that low Se intake provokes a depressed basal activation state of cortical glia, mainly in males, which may result in disrupted homeostatic signals and functions needed to support physiological brain development and plasticity, such as phagocytosis of dead cells, synaptic pruning, the release of trophic factors and cytokines, neural migration guidance [33,51].

The microglial marker CD11b, the receptor for the complement protein C3, is involved in synaptic pruning via microglial phagocytosis, and dysregulation of the complement system and pruning have been suggested to contribute to the onset of neurodevelopmental disorders, such as schizophrenia and autism (see [52] for a rev). The astrocytic marker of activation, GFAP, is also emerging as a player in astrocyte-neuron interactions and in neural plasticity: its deletion in mice was found associated with increased hippocampal neurogenesis and memory extinction, suggesting an increased rate of reorganization of the hippocampal circuitry in these mice [53]. IL-1β, expressed at high levels at times of intense synaptogenesis, during prenatal and postnatal periods [54], modulates synaptic maintenance and plasticity, dendritic complexity, and spine morphology, and is an essential regulator of radial migration of cortical neurons [55]; it can also regulate adult neurogenesis and modulate memory and hippocampal-dependent learning and behavior in a time- and concentration-dependent manner [56,57]. Also the balance between the reciprocally related enzymes iNOS/Arg-1—which compete for l-arginine to produce either nitric oxide or polyamines, respectively—plays physiological roles in brain development [58], besides modulating glial phenotypic polarization from pro- to anti-inflammatory functions during different phases of the inflammation.

In light of this growing knowledge on the neuroplasticity-related functions of immune/inflammatory genes, it becomes clear that a disrupted homeostatic expression of these markers can affect brain physiology and development. Albeit in the hippocampus the effects of suboptimal Se intake were fewer compared to the cortex—as they were limited to CD11b and GFAP dysregulation, the impact on hippocampal developmental trajectory could be no less relevant than in the cortex. Further studies are needed to assess the dynamic of offspring’s gene regulation by low maternal Se intake at earlier and delayed phases of development and the consequences on their behavior.

Notably, whereas the modulation of the expression of a subset of brain inflammatory biomarkers and of GPx activity highlights sex-dependent effects, the behavioral alterations caused by low Se are present in both sexes. During the first weeks after birth, pups exposed through gestation and lactation to deficient or suboptimal Se content diets show typical somatic growth. At the juvenile stage, rats experiencing dietary regimen with suboptimal Se content show hyperactive-like profile when exploring the novel environment represented by the open-field arena, and elevated activity in the Y-maze as indicated by the higher number of entries in the arms and rearing frequency. We could not exclude that later testing of the animals at the attainment of full sexual maturity would have revealed sex differential susceptibility to Se suboptimality in agreement with what was reported for inflammatory biomarkers and GPx activity. Overall, the behavioral domains impaired in Se SubOpt juvenile rats consist mainly in increased exploration and perseveration, mirroring the inability to inhibit inappropriate responding [59]. Such behavioral alterations could be in line with dysfunction of specific areas (i.e., prefrontal cortex and striatum) implicated in attention/working memory and hyperactivity [60]. It is worthy of note that the mesocortical and nigrostriatal dopamine pathways regulating several behaviors including those assessed in our study appear as particularly vulnerable to selenoprotein depletion [61]. As a whole, this set of alterations suggests that neural systems subserving behavioral ontogeny are influenced by the lack of adequate Se intake, in agreement with the increasing evidence from epidemiological and experimental studies implicating Se in critical brain maturation mechanisms [21,22,38,62,63].

The finding that most of the effects are brought about by a suboptimal rather than frankly inadequate Se maternal intake requires specific consideration. An explanation for this apparent paradox could arise from the different impact of the two diets on the actual Se amounts that offspring received during the pre- and post-natal phases. SubOpt offspring had experienced moderately low levels in the prenatal phase and further depleted levels in the postnatal phase, while Def offspring experienced frankly inadequate levels in both phases. Se levels in early maternal milk were indeed similarly low in SubOpt and Def groups, whereas their liver and plasma Se levels at weaning did reveal biologically relevant (although not statistically significant) differences [64] suggesting that, starting with lactation, offspring Se nutrition was equally inadequate in both groups. It is reasonable to hypothesize that the maternal Def diet may have led to a low availability of Se in earlier stages of fetal development compared to the maternal SubOpt diet, thus allowing for adaptation mechanisms and greater resilience in the Def progeny compared to the SubOpt offspring. Further studies are needed to assess the occurrence of alterations, if any, at earlier and delayed stages of development.

## 5. Conclusions

In conclusion, our data add a level of complexity to the emerging picture of the effects of Se maternal nutrition for offspring health, as they point to the possible negative impact of even moderately low maternal Se nutrition during pregnancy and lactation on proper offspring development.

## Figures and Tables

**Table 1:** Se levels in pre-mating female’s plasma and in early milk.

		Plasma Se in Pre-Mating Females (mg/L)		Se in Early Milk (mg/L)	
GROUP		*n*	Median (IQR) ^a^	*n*	Median (IQR) ^a^
Opt	(0.15 mg Se/kg)	5	0.27 (0.02)	6	0.16 (0.05)
SubOpt	(0.04 mg Se/kg)	5	0.24 (0.04)	5	0.05 (0.03) *
Def	(0.02 mg Se/kg)	5	0.24 (0.08)	11	0.05 (0.02) **

Se, selenium; Opt, Optimal; SubOpt, Suboptimal; Def, Deficient. ^a^ Interquartile ranges (75th–25th centile) * *p* < 0.05 and ** *p* < 0.001 vs. Opt.

**Table 2:** Se liver levels in offspring at weaning (PND 23).

GROUP		*n*	M/F ^a^	Median M/F (mg/kg)	Median M + F (IQR) ^b^ (mg/kg)
Opt	(0.15 mg Se/kg)	8	5/3	3.69/3.57	3.63 (0.52)
SubOpt	(0.04 mg Se/kg)	7	4/3	0.70/0.69	0.69 (0.11)
Def	(0.02 mg Se/kg)	8	5/3	0.33/0.33	0.33 (0.06) **

PND, postnatal day. ^a^ M = males, F = females. ^b^ Interquartile ranges (75th–25th centile); ** *p* < 0.001 vs. Opt.

**Table 3:** Se plasma levels in offspring at weaning (PND 23).

GROUP		*n*	Median (IQR) ^a^ (mg/L)
Opt	(0.15 mg Se/kg)	5	0.22 (0.04)
SubOpt	(0.04 mg Se/kg)	5	0.09 (0.03)
Def	(0.02 mg Se/kg)	5	0.04 (0.02) **

^a^ Interquartile ranges (75th–25th centile); ** *p* < 0.001 vs. Opt.

## Data Availability

The data presented in this study are available on request from the corresponding author.

## Supplementary Materials

The following supporting information can be downloaded at: https://www.mdpi.com/article/10.3390/nu14091850/s1, S1: Methods for Plasma, milk, and liver Se determinations; Table S1: Operating conditions used for Se determination by ICP-MS/MS; Figure S1: Body weight (A) and body length (B) in pups assessed on post-natal day (PND) 4, 7, 10 and 12; Figure S2: Anxiety-like behavior in Light/Dark test (A,B) and latency to fall in Rotarod test (C) in juvenile rats.
