# Supplementary material for: Critical Role of Maternal Selenium Nutrition in Neurodevelopment: Effects on Offspring Behavior and Neuroinflammatory Profile
Source: Nutrients. 2022 Apr 28;14(9):1850. doi: 10.3390/nu14091850 (PMC9104078; doi:10.3390/nu14091850)
Supplement: Supplementary file 1 [file nutrients-14-01850-s001.zip › nutrients-1684429-supplementary.pdf]

S1. Methods: Plasma, milk, and liver selenium (Se) determinations

Ultrapure grade water obtained from a Milli-Q Element purification system with 0.22 μm filters was used in the analytical determinations (Millipore, Molscheim, France). The other reagents used were 67–69% *v/v* ultrapure grade nitric acid (HNO_3) (Carlo Erba Reagenti, Milan, Italy) and ultrapure 30% *v/v* grade hydrogen peroxide (H_2O_2) (Sigma Aldrich, St. Louis, MO, USA).

The Se calibration solution and Rh internal standard solution were obtained by dilution from certified standard solutions (High Purity Standards, Charleston, SC, USA 1000 ± 3 μg/mL).

An 8800 ICP-MS Triple Quad spectrometer from Agilent Technologies (Tokyo, Japan) was used to determine total Se. The same sample introduction system described above was used. To eliminate potential spectral interferences on the selenium signal, oxygen was used as a reaction gas in mass shift and the analytical masses were selenium dioxide ion (SeO^+) at mass 94 and 96; the operating conditions are shown in Supplementary Materials Table S1.

Plasma samples were diluted 1:50 with a 0.5% *v/v* HNO_3 solution. Milk samples were digested with HNO_3 and H_2O_2 in a highly efficient microwave system (Ultrawave, Milestone Single Reaction Chamber Microwave Digestion System, Milestone, Bergamo, Italy), with controlled temperature and pressure and an automated cooling cycle, diluted 1:5 before analytical determinations. Liver samples were homogenized and digested with HNO_3 and H_2O_2 in a microwave system (UltraWAVE), diluted 1:20 before analytical determinations.

Total Se quantitative analysis was carried out with external calibration; the calibration range was 0.1–25 μg/L. Rh was used at a concentration of 0.5 μg/L. An ultrapure water sample of the same volume as the samples, collected in the same Eppendorf containers, was used as the procedural blank. The procedural blank (n = 3) was subtracted in the calculation of the sample concentrations.

The analytical determinations were carried out in a clean room environment and, to assess measurement accuracy, analytical quality control procedures were adopted. Seronorm Serum L1 was used as reference material for Se plasma and it was subjected to the same analytical procedure as the samples till the final instrumental determination. The measured value was 0.064 mg/L (standard deviation (s.d.) 0.001 mg/L, n = 3), in good agreement with the certified value of 0.059 mg/L (acceptance range: 0.054-0.065 mg/kg). NIST 1549 (Non-Fat Milk Powder) and BCR 063R (Skin Milk) were used as certified reference materials for Se milk and were subjected to the same analytical procedure as the samples till the final instrumental determination. The measured value for NIST 1549 was 0.11 mg/kg (s.d. 0.01 mg/kg, n = 6), compared to a certified value of 0.11 mg/kg (acceptance range: 0.10-0.12 mg/kg), whereas for BCR the measured value was 0.131 mg/kg (s.d. 0.02 mg/kg, n = 3), compared to an indicative value of 0.129 mg/kg. NIST Bovine liver 1577c were used as certified reference materials for Se liver and were subjected to the same analytical procedure as the samples till the final instrumental determination. The measured value was 2.011 mg/kg (s.d. 0.046 mg/kg, n = 3), in good agreement with the certified value of 2.031 mg/kg (acceptance range: 1.986–2.076 mg/kg).

Table S1. Operating conditions used for Se determination by ICP-MS/MS.

Instrumental Parameters	Operating Conditions
Power RF	1550 W
Nebulizer	Esi PFA-LC
Spray Chamber	Scott PFA inert kit
Nebulizer gas flow	0.82 L min^{-1}
Makeup gas	0.31 L min^{-1}
Nebulizer pump	0.10 rps
Acquisition mode	MS/MS reaction
Reaction gas	Oxygen
Percentage of the reaction gas	20%
Sampling time	30 sec
Time / mass integration	2 sec
Masses selected from Q1	78, 80, 103
Masses selected from Q2	94, 96, 103

Se, selenium; ICP-MS, inductively coupled plasma-mass-spectrometry; MS, mass-spectrometry; RF, radio frequency; Q1 and Q2, quartiles; PFA, perfluoroalkoxy; LC, liquid chromatography; rps, revolution per second.

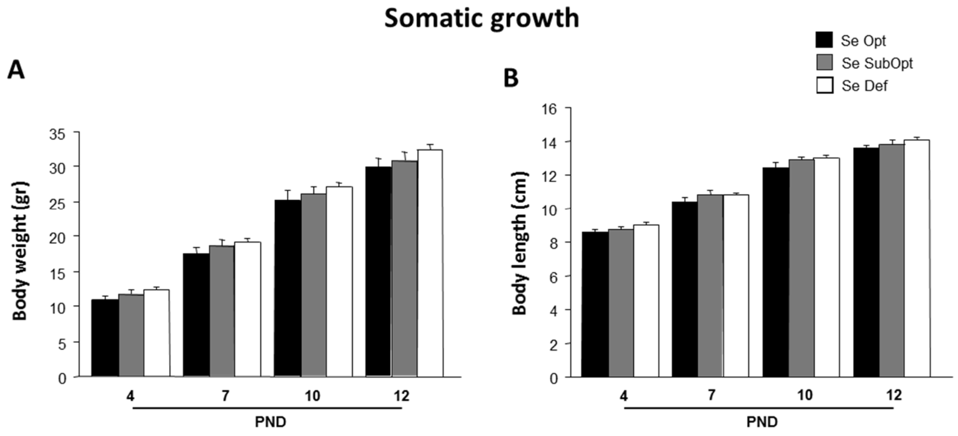

Figure S1. Body weight (**A**) and body length (**B**) in pups assessed on post-natal day (PND) 4, 7, 10 and 12. Se Opt n = 5M, 5F; Se SubOpt n = 6M, 6F; Se Def n = 6M, 6F. Data are sex pooled and represented as mean ± standard error of the mean (SEM). Se, selenium; Opt, Optimal; SubOpt, Suboptimal; Def, Deficient.

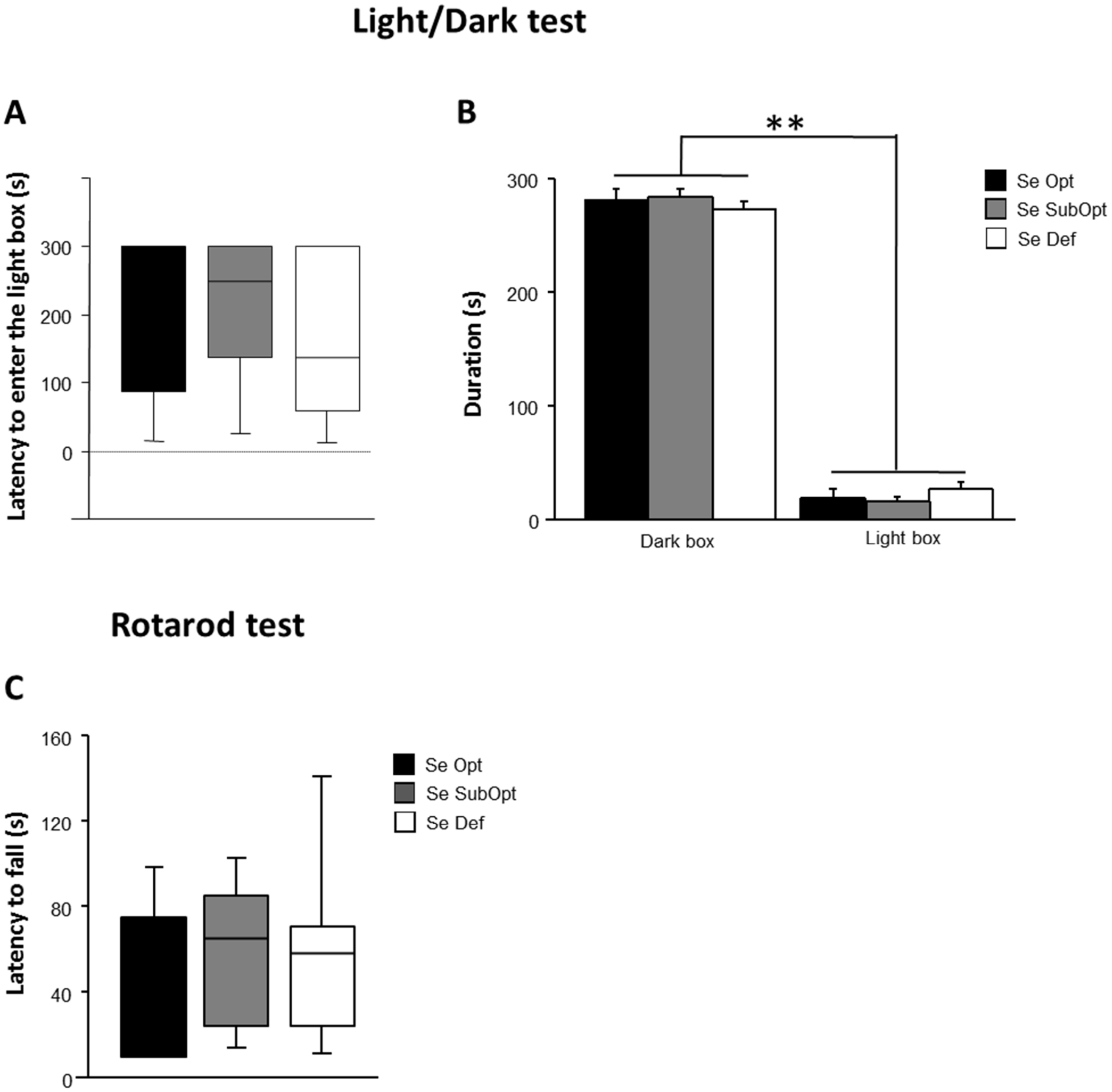

Figure S2. Anxiety-like behavior in Light/Dark test (**A, B**) and latency to fall in Rotarod test (**C**) in juvenile rats. Se Opt *n* = 6M, 6F; Se SubOpt *n* = 7M, 7F; Se Def *n* = 7M, 6F. All data are sex pooled and represented using box plot except for panel B, represented as mean ± SEM. ** < 0.01.
